# Electrically activated quasi-BICs in metasurfaces through atomically thin semiconductors

**DOI:** 10.1038/s41377-026-02451-x

**Published:** 2026-08-26

**Authors:** Ramón Paniagua-Domínguez, José A. Sánchez-Gil

**Affiliations:** Instituto de Estructura de la Materia, Consejo Superior de Investigaciones Científicas, Madrid, Spain

**Keywords:** Nanophotonics and plasmonics, Photonic devices

## Abstract

A new class of electrically tunable nanophotonic devices is presented combining atomically thin 2D materials and metasurfaces supporting quasi bound states in the continuum, with appealing potential applications.

## Abstract (web summary)

Electrically tunable nanophotonic device based on the integration of resonant dielectric metasurfaces supporting quasi bound states in the continuum with atomically thin semiconductors.

## Graphical abstract

**Figure:**
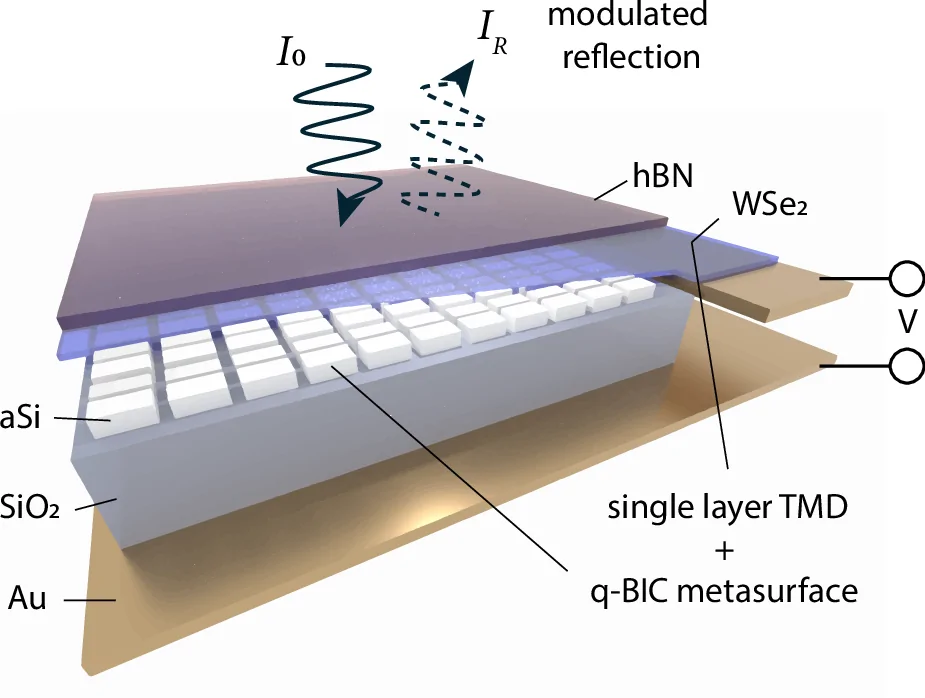

Metasurfaces have attracted a great deal of attention in recent years for their ability to manipulate light as 2D planar devices^1^. A particularly important class of metasurfaces are those recently termed as *non-local*. These are systems able to enhance light matter interactions via high-quality factor collective resonant modes. A subclass of those are metasurfaces supporting Bound states In the Continuum (BICs)^2^, which, due to symmetry mismatch or interference effects, cannot radiate - or receive - light to - or from - the far-field. Currently, a wealth of BIC-supporting metasurfaces have been proposed throughout the electromagnetic spectrum in numerous fields of photonics, and with applications spanning several key areas: sensing and imaging, where BIC’s refractive index sensitivity enables ultra-sensitive biosensing and high-resolution bioimaging^3^; light emission and detection, where BICs can mediate mirror-less lasing^4,5^, strong coupling with excitons for polariton condensation^6,7^, spontaneous emission from quantum emitters^8^; and in many other optical processes and components, which notably includes boosting nonlinear optical processes^9,10^, engineering electromagnetically induced transparency^11^, generating complex quantum states^12^, and advancing chiral photonics and advanced optical device concepts^13,14^.

For many of these applications, active manipulation of BICs and q-BICs is crucial, sparking research on dynamically controlling their high quality-factors^15,16^ (Q-factors), resonance wavelengths^17^, and/or spatial and polarization characteristics^18^. This involves employing materials whose optical properties can be tuned by the application of external stimuli. To name a few: nematic liquid crystals whose orientation is controlled by an external electric^19^ or magnetic field^20^; phase-change materials, which allows for the dynamic (thermal/electrical) tuning of their properties^21^, potentially in a non-volatile fashion; electro-optical materials, such as LiNbO_3_, which enable modulation rates towards the GHz^22,23^; or semiconductors enabling ultra-fast, all-optical control of the q-BIC^15,16^, and where achieving permittivity-asymmetry q-BIC resonances in an otherwise featureless thin film with an intrinsically flat optical response becomes accessible^24^.

While each of these approaches for (q-)BIC tuning has its own advantages and limitations, the seek for novel platforms bringing together the benefits of fast and large modulation depths and electrical addressing for practical applications still goes on. In this regard, the family of two-dimensional (2D) materials^25,26^, spanning insulating hexagonal boron nitride (hBN), semiconducting transition metal dichalcogenides (TMDs), and semimetallic graphene^27^, provides a versatile platform for exploring widely tunable light–matter interactions across a broad spectral range. Recall that room-temperature exciton-BIC polaritons have been demonstrated with TMD metasurfaces, with no active (electric) control though^28^.

In a newly published paper in *Light: Science & Applications*, Ustinov et al. harness this emerging class of materials to put forward an electrically tunable nanophotonic device based on the integration of resonant dielectric metasurfaces with atomically thin semiconductors^29^, specifically 2D-TMDs. As illustrated in Fig. 1, the authors introduce a hybrid nanophotonic platform that combines a WSe₂ monolayer with a dielectric metasurface supporting a q-BIC resonance, enabling active electrical control of optical properties at room temperature. By applying a gate voltage to shift the Fermi level in the monolayer, the authors achieve reversible modulation of excitonic and trionic populations, resulting in a reflectance change of up to ~20% experimentally within the excitonic bandwidth; an effect amplified by strong light–matter coupling through the q-BIC mode. The study demonstrates polarization-dependent behavior, validates the mechanism through a Drude–Lorentz-based conductivity model, and highlights the negligible tuning achievable without the metasurface, underscoring its critical role.

**Fig. 1:**
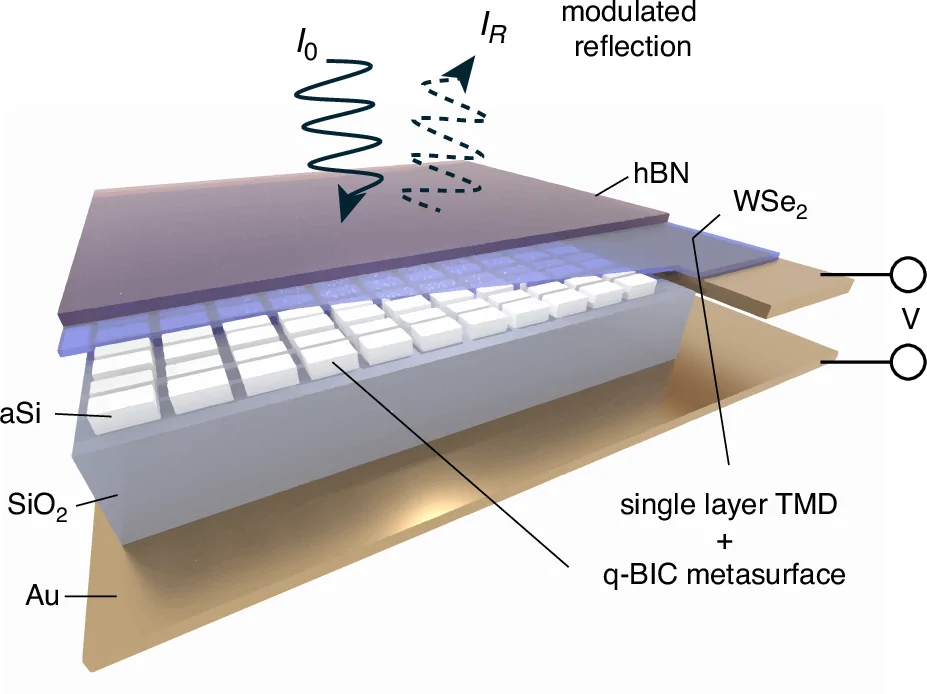
Schematics of the realized electrically tunable nanophotonic device based on the integration of resonant dielectric metasurfaces supporting quasi bound states in the continuum with atomically thin semiconductors

Beyond fundamental insights into exciton–photon interactions, this approach offers a novel route to compact, ultrafast, and reconfigurable optical elements, paving the way for applications in adaptive optical components in analog computing, optical neural networks, and AR/VR systems. Supported by advanced modeling and experimental validation, this work marks a significant step toward programmable planar photonic devices for future technologies.
